# Traditional place-based diets and their effects on healthy and sustainable food transitions: a systematic literature review

**DOI:** 10.1017/S1368980025101274

**Published:** 2025-10-17

**Authors:** Faten Alharbi, Nenad Naumovski, Rosemary Anne McFarlane

**Affiliations:** 1 Health Research Institute, Faculty of Health, University of Canberra, Canberra, Australia; 2 Food Science and Nutrition, Taibah University, Al-Madinah, Saudi Arabia; 3 Discipline of Nutrition and Dietetics, Faculty of Health, University of Canberra, 11 Kirinari Street, Bruce, ACT 2617, Australia; 4 Functional Foods and Nutrition Research (FFNR) Laboratory, Singapore Institute of Technology, 82608 Singapore; 5 University of Canberra Research Institute for Sport and Exercise (UCRISE), University of Canberra, Canberra, ACT 2601, Australia; 6 Department of Nutrition and Dietetics, School of Health Science and Education, Harokopio University, Athens 17671, Greece; 7 Food, Chemistry and Biotechnology Cluster, Singapore Institute of Technology, 828608 Singapore

**Keywords:** Traditional diet, Place-based diets, Carbon footprint, Nutritional quality, Sustainable healthy diets, EAT-Lancet

## Abstract

**Objective:**

Traditional diets are culturally accepted and adapted to local environments, but globalisation has shifted towards unhealthy, unsustainable eating habits. This study aims to assess the literature on the effects of traditional, place-based diets on health and sustainability and examines the suitability of common tools used to evaluate them.

**Design:**

A systematic search was conducted using the PRISMA 2020 guidelines across seven databases (CINAHL, Cochrane Library, MEDLINE, Scopus, Web of Science, PubMed and Google Scholar), and the protocol was registered with PROSPERO (CRD42023445750). The inclusion criteria were traditional place-based diets, studies examining the nutritional, health benefits and sustainability impacts of traditional food consumption, published in English, with no date restriction.

**Results:**

Eleven studies from Spain, Romania, Portugal, Mexico, Chile, Japan, Uganda and India met the criteria. Assessment tools included carbon footprints (*via* LCA), nitrogen footprints, NRF9.3, Nutri-Score and EAT-Lancet; some incorporated qualitative methods. Mediterranean, Atlantic and Japanese diets aligned well with health and sustainability, whereas meat-heavy or nutrient-deficient patterns raised concerns. Most studies relied on standardised tools and secondary datasets, with limited use of region-specific environmental data or qualitative insights. Only one intervention study was identified.

**Conclusions:**

Traditional diets show promise as culturally appropriate models for sustainable and healthy eating. Current tools designed around standardised, reductionist frameworks often fail to capture the complexity of traditional food systems, including local practices, preparation methods and cultural meaning. To better assess traditional diets, future research should develop regionally adapted indicators and integrate quantitative measures with qualitative insights from local communities.

In recent years, contemporary diets, which largely deviate from traditional diets^(1–3)^, have become increasingly unhealthy, placing a substantial burden on public health and environmental sustainability^(2,4)^. According to the UN, the world population will grow to 10·4 billion by the end of the century^(5)^, driving a growing demand for food. This demand is occurring alongside a nutritional transition accelerated by technological advancements, globalisation and westernisation that is linked to rising rates of non-communicable diseases and increased environmental burdens^(4,6)^. According to data from the WHO, approximately one-tenth of the world’s population suffers from hunger, while 43 % of adults are overweight and 16 % are obese^(7)^. Although technological advances have enabled a growing population to be fed, the current food system fails to ensure environmental sustainability, contributing to climate change through the generation of high greenhouse gas emissions (GHGE) and overconsumption of available resources^(8–10)^. About 30 % of GHGE are associated with the food system^(4,8)^ and are projected to rise by an estimated 80 % if these dietary trends are left unchecked^(11)^. There is an urgent need to transition to sustainable, healthy food systems to assist in the reduction of the burden on the environment and improvements in overall public health. This is emphasised by international and national organisations calling for immediate action^(1,2,4,9,12)^ supported by current research evidence^(13–15)^.

Leading organisations have set out frameworks to encourage sustainable and healthy eating in response. The Intergovernmental Panel on Climate Change (IPCC) recommends transitioning towards healthy, sustainable and locally based diets to help mitigate climate change^(2,9)^. The WHO and the FAO of the UN released sixteen nutritional guidelines advocating dietary changes to align with sustainability principles^(16)^. Furthermore, the EAT-Lancet Commission was established to define targets for healthy diets and food production to meet the health and sustainability needs of global populations and the environment^(1)^. The WHO and FAO define a sustainable diet as one that is adequately nutritious, accessible, economically fair and affordable, safe, culturally acceptable, and that minimises the environmental impact of food consumption and production^(2)^. A sustainable diet is necessary to provide food security and nutrition for present and future generations^(2)^. Despite these global initiatives, many challenges remain in their adoption. Sustainable diets also must be tailored to local cultural contexts and populations to be widely applicable and acceptable^(2,9,17)^.

Traditional diets are shaped over centuries and tailored to local environments and cultures, often emphasising whole foods, seasonal and locally sourced ingredients, and diverse plant-based dishes, aligning well with modern sustainability principles^(18–20)^. These diets should be considered for their potential to address global issues from a culturally sensitive perspective, offering viable alternatives to current food systems^(21,22)^.

However, traditional diets lack a clear definition, ranging from indigenous and ancestral diets to local, minimally processed foods. A relatively recent review of twenty-three definitions (1995–2019) found no consensus but identified four common traits: time, place, skills and cultural meaning, with intergenerational knowledge emerging as the most frequent characteristic. Most research is Europe-based and consumer-focused, highlighting the need for clearer, locally grounded definitions^(23)^.

For this review, we adopt a working definition of traditional place-based diets as the locally available foods culturally recognised within a community^(24)^. These foods are specific to a certain place and population, supported by recipes and cooking techniques passed down over generations^(24,25)^. They commonly reflect cultural identity and are associated with happiness, love and social connection^(26)^. Recognising these complexities is critical, as such diversity and cultural embeddedness challenge the use of standardised health and sustainability metrics, which often rely on nutrient composition or environmental footprints without accounting for contextual or cultural dimensions. The Mediterranean diet (MedD) has been the subject of substantial attention in scientific research as a dietary pattern that promotes both health and environmental preservation^(27–29)^. Emphasising the consumption of plant-based foods (vegetables, fruits, legumes and unrefined grains), while also incorporating moderate amounts of meat, fish and olive oil^(28)^. UNESCO’s recognition of the MedD as an Intangible Cultural Heritage of Humanity highlights not only its nutritional value but also the lifestyle and cultural practices embedded within^(28,29)^. Similarly, the traditional Japanese diet, Washoku, which includes nutrient-rich foods such as soyabeans, seaweed, green tea and fish, is also acknowledged by UNESCO for its holistic cultural significance^(21)^. Both diets have demonstrated positive health outcomes and environmentally sustainable practices within their regions^(19,21,30)^. However, there remains a significant gap in the exploration of other traditional diets from different regions, whose diversity and potential benefits are still underexamined.

This review examines how traditional place-based diets have been assessed for health and sustainability in the global literature and evaluates the relevance of common metrics in capturing their cultural and contextual complexity. The goal is to identify effective, evidence-based approaches for promoting sustainable, healthy diets across diverse populations.

## Methods

A systematic review of the literature was conducted to identify all studies examining traditional diets within their cultural contexts as a tool for sustainable and healthy diet transformation. The study protocol was registered with the International Prospective Register of Systematic Reviews (PROSPERO) at the University of York (ID: CRD42023445750).

### Search strategy

**Figure 1.**
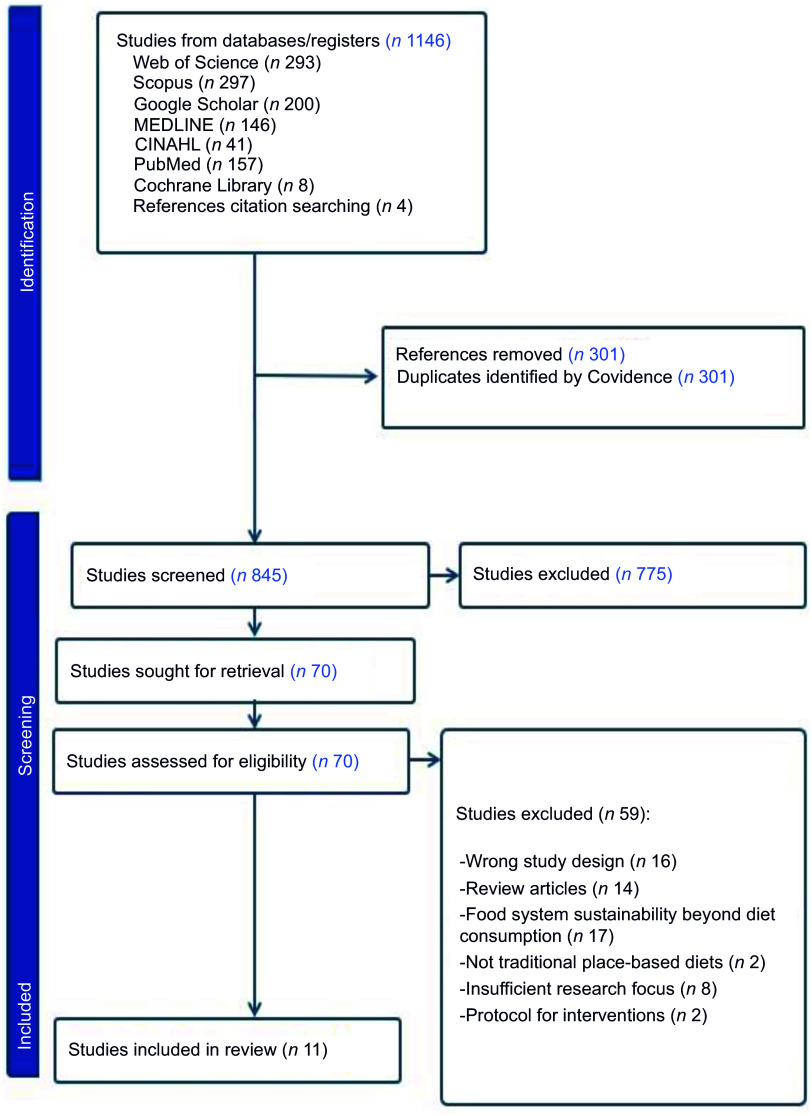
PRISMA flow diagram of the study selection process. The diagram illustrates the identification, screening, eligibility and inclusion stages for articles in the systematic review. PRISMA, Preferred Reporting Items for Systematic Reviews and Meta-Analyses.

This review followed the PRISMA 2020 protocol (Figure 1)^(31)^. Seven electronic databases were searched (CINAHL, Cochrane Library, MEDLINE, Scopus, Web of Science Core Collection, PubMed and Google Scholar) using keywords developed with the Population, Intervention, Comparison, Outcome (PICO) and included key terms such as ((‘traditional diet*’ OR ‘traditional food*’ OR ‘place-based diet*’ OR ‘place-based food’) AND (‘health*’) AND (‘sustainable*’ OR ‘environmentally friendly’ OR ‘EAT-Lancet’)). A search of grey literature was conducted to identify related studies, and reference lists of relevant studies that met the inclusion criteria were reviewed. Literature searches were concluded in June 2024 without a time restriction on publication date. Only articles published in English were included.

### Eligibility criteria

#### Inclusion

All traditional place-based diets were eligible. Included studies examined the health benefits and sustainability impacts of traditional food consumption, including environmental sustainability assessments, but were restricted to diet and food consumption only. Additionally, included studies were required to present the design, implementation, promotion or evaluation of traditional place-based diets to health and sustainability outcomes and to assess these diets against established health and sustainability guidelines. All study designs providing relevant information to the research question, including both grey literature and peer-reviewed original articles, were eligible.

#### Exclusion

Studies were excluded if they examined non-traditional diets or traditional diets outside traditional locations, did not assess the health and sustainability impacts of the diets, focused on traditional diet components or food systems threatened by environmental pressures, assumed sustainability without explicitly addressing it, or examined individual nutrients or food ingredients without considering their broader context. We also excluded studies focusing on farmers or agricultural practices, production and hunting, as well as those studies addressing the loss of traditional diets and related food insecurity due to climate and social change. Reviews, opinion articles, editorials, commentaries, letters to the editor, conference abstracts and publications lacking original research content, or which failed to provide sufficient data for analysis or protocols for interventions were excluded. We found overlaps in sustainability themes beyond this review’s scope, leading to categorised exclusions by two authors (FA and RM).

#### Process of selection and data collection

Following the search strategy, eligible papers were identified and integrated into the Covidence software (https://www.covidence.org), and duplicates were removed^(32)^. The inclusion/exclusion criteria were applied independently by two authors (FA and RM) who screened papers by title and abstract. Any discrepancies were resolved through consultation between the authors and a third author (NN).

#### Data extraction

Data were extracted and organised into a pre-defined table identifying the methods and tools used to assess the nutritional and sustainability impacts of traditional place-based diets and the key findings (Table 1). Considering recent publications and the growing interest in the field, our screening of manuscripts identified various combinations involving traditional diet, health and sustainability concepts. Applying our criteria strictly limited findings to studies focused solely on diet and food consumption.

**Table 1.** Study characteristics, methodology and tools used for articles included in this review of traditional place-based diets as a tool for transforming health and sustainability

Author and year	Traditional diet evaluated [Country]	Study design	Dietary data source	SF and Meth	Nutritional quality and health assessment	Assessment of environmental impacts	Key findings
Oita *et al.*, 2018^(21)^	Traditional Japanese [Japan]	Quantitative observational with four diet scenarios	Food protein supply data (FAO)	SF: Examined the impact of Japanese diet changes on food NF. Meth: Calculated per capita ‘food NF’ for nitrogen emissions from food consumption. Distinguished production (N intake and virtual N factors) and consumption (N intake minus sewage denitrification). Used the N-Calculator method to compare four alternative diets to the 2011 diet (recommended protein, pescetarian, low-NF and traditional Japanese diet).	Based on prior research, health benefits: longevity, cardiovascular health.	NF	A balanced Japanese diet reduces food NF to 12·6 kg N (from 15·2 kg N currently). A Japanese diet with one meat-free day per week reduces NF by more than 20 %. The traditional Japanese diet is a viable low-NF alternative.
Campirano *et al.*, 2023^(33)^	Mexican diet [Mexico]	Quantitative cross-sectional	Health Workers Cohort Study (2004), 1908 adults	SF: Develop a SDS to estimate adherence to the reference diet established by the EAT-Lancet. Meth: SDS was developed using EAT-Lancet guidelines, using cut-off points for nutrient deficiencies and summing component scores on a 0–140 scale.	Nutritional quality assessed via SDS based on FFQ	SDS assessed 14 components (whole grains, fruits, vegetables, dairy products, red meat, poultry, fish, legumes, nuts and fats) and compared compliance with planetary health standards	The Mexican diet averaged 80·5 out of 140 on SDS. Indicates 57·5 % adherence to EAT-Lancet guidelines in Mexico. Highlights the importance of country-specific dietary indices.
Voinea *et al.*, 2020^(34)^	Romanian traditional [Romania]	Quantitative Descriptive Observational	Three traditional Romanian menus (breakfast, lunch and dinner) by chef Sanda Marin.	SF: Assessment of the nutritional quality and sustainability of the Romanian traditional diet. Meth: Nutri-Score algorithm is used to evaluate the traditional Romanian diet based on nutritional and sustainability criteria.	Nutri-Score algorithm: Uses USDA data to assess nutritional quality. Ranking: Colour-coded A (high) to E (low). Negative impacts: Energy, sugars, fats and Na score 0–10 points (N). Positive impacts: Fruits, vegetables, fibre and protein score 0–5 points (P). Final score: Total P subtracted from total N, yielding A-E categories.	Sustainability scores calculate the proportion of plant-derived nutrients (fruits, vegetables and nuts) in comparison with animal-based ingredients.	Most Romanian dishes rated C (average quality), except cabbage salad scored A (high quality). Most sustainable: Cabbage salad (85·84 % vegetables). Least sustainable: Pork stew (mostly animal ingredients, no vegetables). The traditional Romanian diet is unbalanced, with most consumers favouring meat at three meals a day.
Voinea *et al.*, 2020^(35)^	Romanian traditional [Romania]	Qualitative semi-structured interviews	21 Romanians who consume traditional foods	SF: Analysed Romanian traditional food patterns as cultural expressions of sustainability. Meth: 21 Romanians who consume traditional food were interviewed, with data collected on demographic and sociocultural factors. Consumption behaviour. Willingness to adopt more sustainable diets.	Evaluated through consumer interviews, focusing on: Reasons for traditional foods. Regional preferences. Purchasing locations and demographics. Family involvement in cooking. Frequency of traditional dish preparation. Meal/snack frequency, locations and companions. Descriptions of breakfasts, lunches and dinners. Weekday *v*. weekend meal differences. Traditional dish consumption during holidays. Frequency of choosing Romanian cuisine at restaurants.	Evaluation of consumer interviews against author-defined sustainability criteria: Meat consumption concerns. Willingness to substitute meat with plant-based options. Reasons for prioritising local foods. Efforts to minimise food waste. Household waste sorting frequency and methods.	High consumption of meat-based traditional foods. Limited environmental sustainability awareness. Recommend a new sustainable diet model linking tradition, sustainability and health.
Kanter & León Villagra, 2020^(36)^	Traditional Chilean- central Chile LaAraucanía [Chile]	Qualitative participatory	Food-frequency and taste preference questionnaires assessed consumption and taste preferences in Chilean adults (25–45, 46–64 and 65+ years), including First Nations and others	SF: Identification of traditional culinary preparations for subsequent application in the design of healthy and sustainable interventions. Meth: Implemented a toolkit to assess healthy, sustainable traditional diets per ‘*Guidelines for documenting traditional food systems of Indigenous people*’. Cultural domain data collection: Direct observation of traditional cooking methods. Community workshops with a free listing of traditional foods. Pile sort activities in semi-structured interviews. Integrated sustainable diet criteria.	Collect TF data through interviews and workshops. List TF by frequency and cultural significance. Evaluate nutritional value, consumption patterns and taste preferences using a Likert scale and FFQ.	A Sustainable Diet Score was developed using pile sorting. Evaluated traditional dishes based on five criteria: cultural relevance, environmental impact, accessibility, affordability and nutrition. Participants assigned points for each positive trait	Chilean traditional dishes are widely consumed, with regional differences between the Metropolitan Region and La Araucanía. About 600 traditional foods and preparations were identified. A shortlist of 24–27 popular and frequently consumed dishes was created. Traditional diets offer potential for promoting healthy, sustainable eating. Cultural and geographical contexts are key to effective dietary interventions.
Esteve-Llorens *et al.*, 2019^(22)^	Galician Atlantic diet, Northwestern [Spain]	Quantitative observational study	Weekly Atlantic diet plan. Five daily menus reflecting Galician eating traditions. Tailored to 2100 kcal per FAO (2014) guidelines	SF: Analyse the CF and nutritional quality of the Atlantic diet. Meth: CF quantified using Life Cycle Analysis methodology – nutritional quality evaluated using the NRD9.3	Nutritional quality assessed by (NRF9·3).	CF assessed via LCA. Includes household energy consumption and production emissions.	Estimated CF: 21·04 kg CO2eq per person per week (3·01 kg CO2eq per person per d). High nutritional score of 474. Low intake levels of Na, added sugars and saturated fats, staying below recommended limits. CF and nutritional index align with other MedD studies. Combined nutritional and environmental benefits are recommended.
Esteve-Llorens *et al.*, 2020^(37)^	Portuguese diet [Portugal]	Quantitative observational	Nutritional data from Portuguese National Institute of Statistics balance surveys, 2008–2016 (9-year period).	SF: Assess the nutritional quality and environmental impact of the Portuguese diet and propose an EAT-Lancet-based alternative. Meth: Evaluated nutritional quality and calculated the CF (2008–2016). Proposed a healthier, sustainable, lower-calorie diet for Portugal, reducing grains, meat, fats, sugar and potatoes, while increasing legumes, fruit, vegetables, nuts and olive oil.	Nutritional quality assessed by (NRF9.3).	CF assessed via LCA. Includes production, distribution and household activities.	Evaluated Portuguese Dietary Patterns (2008–2016): Average CF: 4·20 kg CO2 eq·inhabitant–1·day–1. Average NRD9.3 score: 371. High CF is linked to energy and livestock consumption. High energetic intake correlates with high CF and low nutritional quality. Proposed Alternative Diet (EAT-Lancet based): Nutritional quality improves by 67 % (NRD9.3 score: 621). CF reduced by 25 % (3·29 kg CO2 eq·inhabitant–1·day–1). Daily energy intake reduced from 3017 to 2764 kcal per capita.
Sáez-Almendros *et al.*, 2013^(27)^	Mediterranean and Spanish [Spain]	Quantitative observational comparative	MedD comes from the new MedD pyramid; they analysed the minimum servings of several food groups^(38)^. (SCP): Based on FAO data and (SCPCS). (WDP): by the US diet using FAO data.	SF: Evaluated GHGE, land use, energy and water of the MedD diet in Spain. Comparison of MedD, Spanish and US diets. Meth: Dietary and environmental data were linked via food group consumption patterns. - Dietary patterns (MedD, SCP and WDP) were based on average consumption. - MedD used minimum servings from the new MedD pyramid^(38)^. - SCP estimated from 2007 FAO sheets (SCPFB) and Household Surveys (SCPCS). - WDP modelled on US data from FAOSTAT. Calculated environmental footprints (GHG, land, energy and water) using LCA data from Spanish^(39)^, EU^(40)^, US^(41)^ and UK^(42)^sources and elsewhere.	Based on prior research, MedD is nutritionally adequate^(43)^/prevents chronic diseases^(44)^.	Analysed GHGE, land use, energy and water consumption. Derived footprints via LCA. Examined phases: production, processing, packaging, transportation and retail.	MedD in Spain reduces GHGE, land use, energy and water consumption significantly. Higher MedD adherence cuts GHGE by 72 %, land use by 58 %, energy use by 52 % and water use by 33 %. The Western diet increases GHGE, land use, energy and water use by 12 % to 72 %. MedD supports food system sustainability and is a cultural and healthy dietary model. Besides health benefits, MDP also offers environmental advantages.
Armes *et al.*, 2024^(45)^	Santal Tribe Diet in Eastern India]	Quantitative comparative nutritional analysis	Two menu templates represent traditional Santal recipes from a Santal cookbook: Kanhu Thali (Winter) Jhano Thali (Late Summer to Monsoon) In Santal culture, ‘Thali’ denotes three daily meals: Morning – Day Evening	SF: Evaluate traditional Santal recipes against dietary standards to identify improvements in nutrition and sustainability. Meth: Assessed nutritional adequacy of Santal recipes. Compared menu templates with EAT-Lancet guidelines.	Used Nutritics Professional Premium software for nutritional analysis of Santal recipes. Analysed energy content, macronutrients and micronutrients. Compared results with EAT-Lancet guidelines.	Assessed environmental impact by comparing Santal menus with EAT-Lancet guidelines. Focused on plant-based diets, reducing animal products. Identified alignment and divergence from sustainability practices.	Santal menus align with EAT-Lancet guidelines by emphasising whole grains, starchy vegetables and plant-based proteins from legumes. Central staples include rice, wheat, maize and indigenous fish/snails, with limited animal-based protein and dairy products. Average intake: 453·6 g of vegetables (exceeds Indian RDA) and 97 g of fruit (approaches EAT-Lancet recommendations). Santal diets avoid animal-based protein and dairy products, relying on indigenous fish and snails. Low consumption of high-quality protein like meat and dairy products reflects cultural practices and availability.
Cambeses *et al.*, 2024^(46)^	Galician Atlantic diet, Northwest [Spain]	Randomised clinical trial intervention	Captured through a 3-d food diary to assess individual nutritional intake.	SF: Assess MetS incidence and CF effects of a 6-month intervention based on the Galician Atlantic Diet. Meth: Study of 231 families (270 adults intervention group, 248 control group). The intervention group received tailored dietary counselling, including three nutrition sessions, cooking classes, written materials and regular food baskets with Atlantic diet items. The control group maintained the usual lifestyle throughout the study.	Measured metabolic variables: Waist circumference TAG levels HDL cholesterol Blood pressure Fasting glucose level	LCA was used to calculate each participant’s dietary CF.	The 6-month intervention reduced MetS risk by 42 % compared with the control. No significant reductions in high blood pressure, hypertriglyceridemia or hyperglycaemia were observed in either group. Food consumption did not significantly reduce environmental impact between groups. Baseline and 6 months: Control group: Baseline: Mean (sd), 3·71 (1·55) kg CO2eq/person/d. After 6 months: Mean (sd), 3·56 (1·50) kg CO2eq/person/d. Intervention group: Baseline: Mean (sd), 3·60 (1·44) kg CO2eq/person/d. After 6 months: Mean (sd), 3·38 (1·39) kg CO2eq/person/d.
Auma *et al.*, 2019^(47)^	Ugandan Diet [Uganda]	Quantitative A cross-sectional survey	The Uganda Food Consumption Survey = 957 women (15–49 years) multi-stage cluster sampling.	SF: Explore dietary transitions and environmental sustainability implications among Ugandan women. Meth: Utilised PCA to identify dietary patterns from Uganda Food Consumption Survey data. Recorded 531 food items into 35 categories based on culinary use, tradition and environmental impact.	Used a single 24-h recall and PCA to identify dietary patterns. Categorised food intake to assess essential nutrient levels.	Estimated from GHGE data. Foods were categorised as: low impact (GHGE < 1·0 kgCO2eq/kg), medium impact (GHGE 1·0–4·0 kgCO2eq/kg), or high impact (GHGEs >4·0 kgCO2eq/kg).	The traditional Ugandan diet is characterised by high-fat content and medium environmental impact GHGE 1·0–4·0 kgCO2eq/kg. It emphasises fats, oils, proteins and conventional plant foods, potentially high in saturated fats, suggesting suitability for Ugandan women with appropriate quantities of red meat added. The traditional diet exhibits signs of nutritional transition by consuming a high-fat intake despite deep cultural roots.

SF, study focus; Meth, methodology; TF, traditional food; NF, nitrogen footprint; CF, carbon footprint; LCA, life cycle assessment; GHGE, greenhouse gas emissions; SDS, Sustainable Dietary Score; USDA, United States Department of Agriculture; NRD9.3, Nutrient-Rich Dietary Index; MedD, Mediterranean diet, SCP, Spanish diet, SCPCS, Spanish Ministry Surveys, WDP, Western diet, MetS, metabolic syndrome, RM, metropolitan region, AR, Region of La Araucanía, PCA, principal component analysis.

#### Assessment of quality

Due to the diverse and heterogeneous research methodologies employed, we encountered challenges in evaluating the quality of the studies. Consequently, direct comparisons between the studies were not feasible. Given this heterogeneity in study designs, populations and methods, a narrative synthesis approach was used to integrate and interpret the findings.

## Results

### Study selection

Studies were identified and selected as shown in the PRISMA flow diagram (see Figure 1). An initial search yielded 1146 results. After removal of duplicate articles and title and abstract screening, seventy full-text eligibility were assessed by two researchers (FA and RM). This process led to the inclusion of eleven studies^(21,22,27,33–37,45–47)^.

### Study characteristics

This review examined traditional diets across populations in eight countries: Spain.^(22,27,46)^, Romania^(34,35)^, Chile^(36)^, Japan^(21)^, Portugal^(37)^, Mexico^(33)^, Uganda^(47)^ and India^(45)^ (Table 1).

Only two studies^(35,36)^ used qualitative methods with specific populations: the Chilean ethnic group^(36)^ and Romanians^(35)^. Five studies utilised secondary data sources and included Portuguese food balance surveys.^(37)^, Japanese FAO data^(21)^, Mexican Health Workers from the Cohort Study^(33)^, Spanish FAO data and Spanish Ministry Surveys. Two studies examine minimal servings from the new Mediterranean Pyramid (MDP)^(27)^ and Ugandan food consumption data from a nationally representative survey^(47)^. Others used traditional recipes^(34,45)^, weekly diet plans^(22)^ and clinical trial analyses^(46)^.

The studies focused on different outcomes: environmental impact only (*n* 2)^(21,27)^, both nutrition and environment (*n* 4)^(22,34,37,47)^, health and environmental impact (*n* 1)^(46)^, cultural and sustainability insights (*n* 2)^(35,36)^, and alignment with global guidelines from the EAT-Lancet Commission (*n* 2)^(33,45)^. Only one was a randomised controlled trial^(46)^, evaluating a 6-month Atlantic diet intervention for its effects on metabolic syndrome (MetS) and carbon footprint (CF). The rest used observational or cross-sectional designs.

The environmental impact of traditional diets was assessed using indicators such as GHGE, carbon and nitrogen footprints (NF), land use, energy, and water consumption. Four studies^(22,27,37,46)^ used life cycle assessment (LCA): Two in Spain evaluated the Atlantic diet’s CF^(22,46)^, one in Portugal assessed the Portuguese diet’s CF^(37)^ and another in Spain analysed the GHGE, land energy and water use of adhering to MedD^(27)^. Additionally, a Ugandan study classified foods by GHG impact^(47)^, while a Japanese study focused on the NF^(21)^.

Nutritional quality and health outcomes were assessed using various tools. Three studies^(22,34,37)^ evaluated dietary nutritional quality, two used the Nutrient-Rich Diet (NRD9.3) score^(22,37)^ and one applied the Nutri-Score algorithm^(34)^. A randomised controlled trial^(46)^ evaluated the Atlantic diet’s effects on MetS, offering potential evidence of health impact.

These studies provided primary analyses of health and sustainability, with four studies assessing both environmental impact and nutritional quality or health outcomes^(22,37,46,47)^. While two studies^(21,27)^ focused solely on environmental aspects, they referenced health outcomes indirectly. The Japanese study measured only the NF^(21)^, and the Spanish study compared the environmental performance MedD to Spanish and US diets^(27)^. Other studies aligned traditional diets with EAT-Lancet guidelines^(33,45)^. In India, nutrient intakes were compared with EAT-Lancet targets^(45)^, while in Mexico, a Sustainable Dietary Score (SDS) was developed^(33)^. Qualitative approaches were also used in Chile and Romania to explore cultural values and sustainability through cultural domain analysis^(36)^ and semi-structured interviews^(35)^ (Table 1).

### Environmental impact of traditional diets

The Atlantic, MedD, Ugandan and Japanese diets were all assessed as sustainable, showing lower environmental impacts such as reduced CF, GHGE and NF compared to the contemporary Western diet. However, the extent varied by dietary pattern and context.

The Atlantic diet, assessed in northwestern Spain, was associated with a CF of 3·01 kg CO₂eq/d in one observational study^(22)^. A clinical trial in the same region found a small, non-significant reduction of −0·17 kg CO₂eq/d (from 3·71 to 3·38 kg CO₂eq/d) after 6 months (*P* = 0·24)^(46)^. In Portugal, the current national diet had a higher CF of 4·20 kg CO₂eq/d, but modelling an EAT-Lancet-adapted version of the Portuguese diet reduced CF by almost 25 %, to about 3·29 kg CO₂eq/d^(37)^, indicating potential synergies between global dietary recommendations and local adaptations of traditional eating patterns.

The MedD in Spain was associated with significant environmental benefits, including reductions in GHGE (72 %), land use (58 %), energy consumption (52 %) and water use (33 %), while Western diets increased these impacts^(27)^. The Japanese study found that incorporating a weekly meat-free day into a traditional dietary pattern reduced NF by over 20 %, from 15·2 to 12·6 kg N/week^(21)^. Similarly, in Uganda, traditional plant-based diets were categorised as having a medium environmental impact (GHGE 1·0–4·0 kg CO₂eq/kg), notably more sustainable than high-impact, animal-based diets exceeding 4·0 kg CO₂eq/kg^(47)^. Across the included studies, traditional plant-forward diets consistently showed lower environmental impacts than animal-based or Western patterns, which may further support long-standing proposals of their benefits.

### Nutritional quality and health evaluation

The three traditional diets of the Atlantic, Mediterranean and Japanese regions were assessed as being healthy. In northwestern Spain, the traditional Atlantic diet achieved an NRD9.3 score of 450^(22)^. A 6-month randomised controlled trial further demonstrated a reduction in the incidence of MetS (defined as a cluster of conditions that increase the risk of heart disease, stroke and T2DM) in the intervention group compared to controls (2·7 % *v.* 7·3 %, relative risk = 0·32)^(46)^. Significant improvements were reported in waist circumference, central obesity risk and HDL-cholesterol, although no significant changes were observed in blood pressure, TAG or fasting glucose levels^(46)^. The Portuguese study found a 67 % increase in NRD score (from 377 to 621) when following a low-calorie diet aligned with EAT-Lancet guidelines^(37)^. In Spain, the MedD was evaluated through secondary data and found to be nutritionally adequate, with evidence supporting its role in chronic disease prevention^(27)^. Moreover, the Japanese study indicated that adhering to the traditional Japanese diet was associated with positive health outcomes, such as extending lifespan and reducing the incidence of type 2 diabetes mellitus (T2DM) and heart disease^(21)^.

Not all traditional diets meet health standards. Two studies found the traditional Romanian diet unhealthy, citing high meat consumption as a contributing factor.^(34,35)^. However, high meat consumption alone does not determine a diet’s healthiness. The Romanian diet also lacks sufficient vegetables, fibre and other essential nutrients, which contribute to its lower nutritional quality.^(34)^

One study found that most traditional Romanian dishes were rated ‘C’ on the Nutri-Score scale, reflecting average nutritional quality, largely due to frequent consumption of meat-heavy meals three times daily and insufficient plant-based components^(34)^. A qualitative study further reported that daily meat consumption is common in Romania and that there is low acceptance of plant-based diets among the population^(35)^. Both studies highlight the diet’s low vegetable intake and high reliance on animal-based foods, recommending improvements to enhance both health and sustainability^(34,35)^.

### Traditional diet and alignment with global standards EAT-Lancet

Two studies^(33,45)^, evaluated traditional diets against the EAT-Lancet Commission’s reference diet, focusing on nutritional components rather than environmental impact measures. In Mexico^(33)^, a SDS was developed based on the EAT-Lancet framework, incorporating fourteen food components. The average score was 80·5 out of 140, indicating 57·5 % adherence to EAT-Lancet guidelines among adults in the Health Workers Cohort Study^(33)^. In India^(45)^, traditional meals of the Santal tribe partially aligned with EAT-Lancet’s plant-based recommendations but lacked animal protein and dairy products, a divergence driven by cultural practices and geographic factors that deviates from both Indian dietary recommendations and EAT-Lancet guidelines^(45)^. Traditionally, people in the Santal tribe avoid dairy products, in contrast to the Indian recommendations of 300 g per d^(48)^ and the EAT-Lancet guidelines of 250 g per d^(1)^.

### Qualitative insights: cultural and sustainable dimensions of traditional diets

Two qualitative studies^(35,36)^ provided insights by engaging populations to explore personal experiences, regional preferences and sustainability perceptions of traditional foods. In Chile^(36)^, a study used an adapted version of the *‘Guidelines for Documenting Traditional Food Systems of Indigenous People*’^(49)^ to evaluate sustainable traditional diets^(36)^. Originally designed to document traditional food, this toolkit was expanded to assess entire culinary preparations and ingredients, identifying culturally significant foods for sustainable health interventions. The sustainability of dishes was calculated based on cultural suitability, nutritional sufficiency, accessibility, economic fairness and environmental impact^(36)^. The study highlighted diverse traditional preparations, particularly vegetable-based dishes, to guide healthy, sustainable interventions^(36)^.

In Romania^(35)^, a qualitative study identified that participants were highly positive about traditional dishes, mainly due to their cultural significance, evoking memories of childhood and a sense of pride. These foods are highly regarded for their authenticity, nutritional value, freshness and taste, although overconsumption of meat was identified as a challenge to sustainability^(35)^. These qualitative findings highlight the depth of cultural knowledge and lived experiences, offering dimensions of sustainability often overlooked in standardised dietary assessments.

## Discussion

To the best of our knowledge, this is the first systematic review to examine how traditional place-based diets contribute to both health and environmental sustainability while critically evaluating the methodological limitations of current assessments. While traditional diets are often assumed to be inherently beneficial, our findings show this is not always the case. Mediterranean, Atlantic and Japanese patterns exhibit the alignment of nutritional quality with environmental sustainability, whereas Romanian^(34,35)^ and the Indian Santal diets^(45)^ highlight risks of environmental burden or nutritional gaps. These contrasts demonstrate that the sustainability of traditional diets cannot be assumed but requires context-specific and critical assessment.

We note a lack of standardised methods to jointly assess nutritional and environmental adequacy. Although tools like NRD9.3, Nutri-Score, LCA and EAT-Lancet frameworks were widely applied across the included studies, their relevance for capturing the cultural and ecological complexity of traditional diets remains contested. While LCA is widely recognised, most dietary studies focus narrowly on CF or GHGE. These are important climate metrics and are often used as proxies for other impacts (e.g. acidification, eutrophication)^(50,51)^ but can oversimplify food system impacts, overlooking biodiversity loss, soil carbon depletion and food waste^(52,53)^. A review of 113 sustainable diet studies found that GHG were the most frequently used indicator (63 %), followed by land use (28 %) and energy use (24 %)^(54)^.

Additionally, inconsistencies in system boundaries further reduce comparability: some studies assess the full life cycle from production to consumption, while others omit packaging, transport or waste^(22,37)^, potentially underestimating impacts^(50)^. Inconsistent definitions and labelling of similar LCA metrics, along with single-indicator approaches, can distort results or shift impacts between stages or regions^(53)^. These inconsistencies hinder meaningful comparison of traditional diets across settings^(50)^.

Another challenge is the mismatch between standardised LCA indicators and the localised nature of traditional food systems. For instance, only one MedD study assessed multiple indicators, but it relied on generic LCA databases, reducing contextual accuracy^(27)^. Similarly, a study on Ugandan diets^(47)^ reported moderate environmental impacts but relied on global data, reducing its local validity. Such issues often stem from data constraints but undermine ecological specificity and cultural sensitivity, both of which are essential when evaluating traditional diets^(27,47)^.

Ideally, environmental assessments should draw on region-specific data that reflect local agricultural practices, production methods and consumption patterns, including home preparation and waste^(55)^. Until such data are widely available, LCA-based conclusions about traditional diets should be interpreted with caution.

The NF was used in one study assessing the traditional Japanese diet, which found that greater adherence could significantly reduce nitrogen emissions^(21)^. Unlike CF, NF captures reactive nitrogen losses across the food system mainly from fertiliser use and nitrogen-fixing crops, which contribute to air and water pollution, as well as climate change through nitrous oxide (N₂O), a greenhouse gas nearly 300 times more potent than CO₂^(21,56)^. Globally, agriculture accounts for approximately 75 % of N₂O emissions^(55)^.

Originally designed for individuals, NF now applies to institutions worldwide and highlights the environmental cost of protein-rich, fertiliser-intensive diets^(57)^. However, these models typically rely on industrial datasets, often overlooking low-input, seasonal food systems typical of traditional diets. As a result, applying NF without localised data may misrepresent the true environmental footprint of traditional practices.

This limitation is not unique to NF. Most environmental assessment tools, including LCA, draw heavily from datasets based on large-scale, high-input agricultural systems in high-income countries^(58)^. For instance, the Poore and Nemecek database ^(58)^, though comprehensive, primarily reflects industrial farm data. Consequently, traditional, low-impact diets remain underrepresented, and the cultural and ecological specificity of traditional food systems is often ignored. Without localised adaptations, integrated carbon-nitrogen tools risk undervaluing the sustainability potential of traditional diets.

The EAT-Lancet dietary guidelines^(1)^ provide a universal reference diet within planetary boundaries, aiming to limit GHGE to 5 gigatonnes of CO_2_-equivalent per year, nitrogen application to 90 teragrams per year, phosphorus application to 8 teragrams per year and freshwater use to 2500 km^3^ per year, achieving an extinction rate of ten species years of extinction and reducing cropland use to 13 million km^2^^(1)^. Even though these global sustainability benchmarks are valuable, they face challenges when applied across diverse populations. For instance, the Portuguese study reported a reduction in CF when diets were adjusted according to these guidelines^(37)^. In contrast, studies in Mexico and India showed varied outcomes: the Mexican diet met only 57·5 % of the targets^(33,45)^. While the Santal tribe’s diet in India aligned with the sustainability criteria for plant-based foods but lacked animal proteins and dairy products, raising concerns about nutrient adequacy, especially for iodine and vitamin D in an already deficient population^(45)^. These examples underscore a key limitation: EAT-Lancet targets may not fully reflect local nutritional needs, food availability or cultural dietary patterns. Its one-size-fits-all approach may not fully reflect the diversity of traditional dietary practices shaped by cultural, ecological and economic contexts.

The NRF9.3 Index is a widely recognised measure of diet quality, balancing nine nutrients to encourage with three to limit (added sugar, Na and saturated fat). Despite being identified as the most frequently used nutritional quality tool in a scoping review of eighty-two indicators^(50)^, it appeared in only two studies in this review^(22,37)^.

The application of NRF9.3 has additional relevance for sustainability research, as higher scores have been linked to lower GHGE and alignment with high-quality traditional diets^(22,59)^. Its ability to measure nutrient density independently of energy intake supports cross-study comparability^(50)^.

However, the use of NRF9.3 in diverse cultural contexts still remains limited. The application of NRF9.3 may overlook nutrient priorities shaped by local deficiencies, food preparation methods or traditional food combinations. As Drewnowski notes, nutrient profiling models were developed for high-income settings to address obesity, penalising energy-dense foods while ignoring their micronutrient value^(60)^. Applied uncritically in low-income countries, such models risk undervaluing culturally important foods rich in Ca, Fe or high-quality protein. This underscores the need for culturally adapted indices over unmodified global metrics^(60)^. Misalignment between traditional diets and global standards may reflect limitations of the tools rather than shortcomings in the diets themselves. Standardised tools like NRF9.3 and Nutri-Score rely on a reductionist model, focusing on isolated nutrients while ignoring synergistic effects of whole foods, preparation methods and ecological context^(61,62)^. In studies by Fardet and Rock^(63)^ and Monteiro *et al.*^(64)^, traditional diets often feature minimally processed foods, bioactive synergies and seasonal diversity. Nutrient profiling tools that overlook food matrix effects and cultural preparation methods risk undervaluing traditional diets, sometimes ranking ultra-processed foods such as sweetened cereals above nutrient-dense staples like eggs and whole milk, leading to misclassification of diet quality^(62)^.

Nutri-Score, while effective for packaged foods in Europe, depends on per 100 g nutrient data and does no account for mixed dishes or home-prepared meals. Even in France, adaptations were needed for foods such as cheeses and fats to align with national guidelines, showing the need for cultural adjustments^(61)^. Nutrient-based tools may misrepresent traditional diets unless mixed dishes are disaggregated into their components. The Saint Kitts and Nevis National Individual Food Consumption Survey (NIFCS) showed that recipe disaggregation changed key food group estimates,^(65)^. Likewise, analysis of Australia’s 2011–12 National Nutrition and Physical Activity Survey (NNPAS) demonstrated that breaking down composite dishes improved the accuracy of meat, poultry and fish intake estimates^(66)^, underscoring the need for local ingredient data. Without cultural adaptation and a local data, nutrient-based tools may misrepresent traditional diets and overlook their true value.

Case studies from Uganda^(47)^, Japan^(21)^, Romania^(34,35)^ and India^(45)^ illustrate how traditional diets are shaped by local environments, nutrient needs and cultural norms. In Uganda, moderate environmental impacts coexisted with a need for higher meat intake among nutritionally vulnerable groups, especially women of reproductive age^(47)^. In Japan, a minor change of one meat-free day per week reduced NF without compromising nutritional adequacy^(21)^. In Romania, meat-centred traditions posed barriers to sustainability^(34,35)^. While the plant-based diet of the Santal tribe in India failed to meet micronutrient needs, raising concerns about iodine deficiency^(45)^.

These examples highlight the importance of flexibility and cultural sensitivity in dietary recommendations. WHO/FAO and the World Cancer Research Fund guidelines advise limiting red meat to < 71 g/d or 0·5 servings daily^(67)^, but such recommendations must be adapted to population-specific nutritional vulnerabilities. For instance, a Romanian survey (2021–2022, *n* 1053)^(68)^ showed high animal product intake (71 %) and low consumption of fruits, vegetables, nuts and fish (77–81 %), raising health and sustainability concerns^(67)^.

Modifying traditional diets to balance global health and sustainability standards while respecting cultural practices can be beneficial. Adapting traditional diets to meet health and sustainability goals can be beneficial, but changes should be cautious and culturally sensitive. While reducing meat and dairy products may lower environmental impact, these foods often supply Ca, Fe vitamin B_12_ and Zn^(69,70)^. Plant-based diets with moderate meat intake can offer environmental benefits^(71)^, but adequacy depends on local nutrient needs^(71)^.

This review aligns with previous research interest in adhering to MedD patterns. Although MedD is already extensively studied and therefore did not feature prominently here, its dual benefits shared with the Atlantic diet are evident in the NRD9.3 scores, which indicate high nutritional quality alongside a low CF^(22,52)^. Similar studies^(28,52)^ suggest that this stems from their common emphasis on abundant fruits, vegetables, olive oil and fish, combined with simple cooking methods such as boiling and braising. The Atlantic diet differs from the MedD mainly in its stronger focus on local and seasonal foods^(22,52)^ yet both serve as practical examples of how to balance nutritional quality with environmental sustainability^(22,27,46)^. These predominantly plant-based diets, which limit meat consumption, provide diverse nutrient profiles while demonstrating potential for reducing CF and improving diet quality^(21,22,52)^.

In our synthesis of studies assessing both nutritional quality and CF, we observed a consistent inverse relationship: higher diet quality was associated with lower CF. This finding is consistent with other reviews^(52)^ that underscore the environmental advantages of the Atlantic and MedD diets. In addition, the Japanese diet, rich in fish, seaweed, vegetables, soya products, green tea and fruit, combines balanced, nutrient-dense eating with a low NF, offering a culturally distinct model of health and sustainability^(21)^.

A fundamental limitation of standardised tools is their lack of connection to the lived realities of those consuming traditional diets. Secondary or aggregated data can mask intra-cultural differences, making detailed, population-level data essential. Qualitative research can uncover cultural, generational and practical dimensions of food systems, as shown in studies from Romania and Chile^(35,36)^. Combining quantitative and qualitative methods, supported by local expertise, is essential to capture intra-cultural differences and guide sustainable, culturally relevant dietary transitions.

### Strengths and limitations

A key strength of this review is its broad scope, enabling a comprehensive global search and critical evaluation of traditional dietary patterns to health and sustainability. It assesses commonly used tools (NRF9.3, Nutri-Score, LCA and EAT-Lancet) and highlights their limitations when applied to traditional diets.

However, several limitations should be acknowledged. First, limitations of this review include the availability of relevant literature, which may have constrained the breadth of evidence identified. In addition, the substantial variation in methods and indicators across studies reduced comparability and prevented the application of a consistent quality appraisal framework.

Second, limitations of the studies reviewed were also evident. Many investigations modelled meals or weekly menus from FAO guidelines, food pyramids or traditional recipes, which may bias results towards healthier dietary patterns and reduce alignment with real-world consumption. Furthermore, heavy reliance on secondary or global datasets, particularly for LCA and NF analyses, reduced contextual accuracy. Region-specific evidence was limited. Studies were either qualitative or quantitative, but none used mixed-methods to capture the full complexity of traditional diets. No study measured actual consumption of traditional foods or assessed dietary change after interventions.

Ongoing trials, such as a sustainable psycho-nutritional intervention currently underway in Mexico^(72)^ as well as the DELICIOUS Project, a five-country school-based intervention promoting MedD adherence and sustainability education^(73)^, signal growing interest in this field; however, the information available at present is limited to study protocols.

### Future directions

We recommend future work to develop culturally tailored, mixed-method approaches that integrate quantitative indicators with local knowledge, use region-specific datasets, include the voices of local communities and researchers, and expand environmental metrics beyond greenhouse gases. Such approaches will enable more accurate, context-relevant assessments and guide policies that protect and promote traditional diets.

### Conclusion

Traditional place-based diets tailored to local environments have the potential to address both health and sustainability challenges, but not all such diets meet the criteria for health or sustainability.

In many cases, perceived shortcomings reflect the limitations of assessment tools rather than the diets themselves. Standardised nutrient-based or environmental metrics often overlook the cultural, nutritional and ecological complexity of traditional diets. Assumptions about their healthfulness or sustainability should therefore be tested against local nutritional needs, food access and lived realities.

Traditional diets are dynamic and must be evaluated within their social and environmental context. Ideally, desktop evaluation of historical diets should be replaced with regionally adapted evaluations that reflect local food systems, preparation methods and cultural practices. Engaging local researchers and communities would improve accurate and respectful evaluation. A comprehensive approach combining quantitative metrics with qualitative insights into cultural meaning and everyday practices is recommended to fully capture the potential health and sustainability value of traditional diets.
